# Supplementary material for: Resolving the full spectrum of human genome variation using Linked-Reads
Source: Genome Res. 2019 Apr;29(4):635–45. doi: 10.1101/gr.234443.118 (PMC6442396; doi:10.1101/gr.234443.118)
Supplement: Supplemental Material [file supp_gr.234443.118_Supplemental_longranger-2.2.zip › longranger-2.2.tar.bz2!/lr2.2-public/lib/stan/cmdstan/stan/doc/api/html/version_8hpp_source.html]

Stan: src/stan/version.hpp Source File


|  |  |
| --- | --- |
|  | Stan  2.5.0  probability, sampling & optimization |


- Main Page
- Related Pages
- Namespaces
- Classes
- Files

- File List
- File Members

All Classes Namespaces Files Functions Variables Typedefs Enumerations Enumerator Friends Macros Pages

- src
- stan

version.hpp

Go to the documentation of this file.

1 #ifndef STAN\_\_VERSION\_HPP

2 #define STAN\_\_VERSION\_HPP

3

4 #include <string>

5

6 namespace stan {

7

9  const std::string MAJOR\_VERSION = "2";

10

12  const std::string MINOR\_VERSION = "5";

13

15  const std::string PATCH\_VERSION = "0";

16

17 }

18

19 #endif

stan::MINOR\_VERSION

const std::string MINOR\_VERSION

Minor version number for Stan package.

**Definition:** version.hpp:12

stan::PATCH\_VERSION

const std::string PATCH\_VERSION

Patch version for Stan package.

**Definition:** version.hpp:15

stan::MAJOR\_VERSION

const std::string MAJOR\_VERSION

Major version number for Stan package.

**Definition:** version.hpp:9


---

[ Stan Home Page ]

*© 2011–2014,
Stan Development Team.*
